# Efficacy and safety of ambrisentan in Chinese patients with connective tissue disease-pulmonary arterial hypertension: a post-hoc analysis

**DOI:** 10.1186/s12872-020-01591-1

**Published:** 2020-07-17

**Authors:** Mengtao Li, Zhi-Cheng Jing, Yang Li, Yong Huo, Zaixin Yu, Gangcheng Zhang, Ping Zhu, Jinming Liu, Qiushang Ji, Bingxiang Wu, Jinhua Zhong, Pingping Wang, Wenjing Zhu, Xiaofeng Zeng

**Affiliations:** 1 Department of Rheumatology, Peking Union Medical College Hospital, Peking Union Medical College & Chinese Academy of Medical Sciences, National Clinical Research Center for Immunologic Diseases, Ministry of Science & Technology, Key Laboratory of Rheumatology and Clinical Immunology, Ministry of Education, Beijing, China; 2 Key Lab of Pulmonary Vascular Medicine & FuWai Hospital, State Key lab of Cardiovascular disease, National center for Cardiovascular disease, Department of Cardiology, Peking Union Medical College Hospital, Chinese Academy of Medical Sciences & Peking Union Medical College, Beijing, China; 3 2nd Affiliated Hospital of Harbin Medical University, Harbin, China; 4 1st Affiliated Hospital of Peking University, Beijing, China; 5 Xiangya Hospital Central-South University, Changsha, Hunan China; 6 Wuhan Asia Heart Hospital, Wuhan, Hubei China; 7 1st Affiliated Hospital of the Fourth Military Medical University, Xi’an, Shaanxi China; 8 Shanghai Pulmonary Hospital, Tongji University, Shanghai, China; 9 Qilu Hospital, Shandong University, Jinan, Shandong China; 10 GlaxoSmithKline (China) R&D Company Limited, Shanghai, China

**Keywords:** Ambrisentan, Chinese, Exercise capacity

## Abstract

**Background:**

The efficacy and safety of ambrisentan has been previously evaluated in Chinese patients with pulmonary arterial hypertension (PAH). This post-hoc analysis assessed the efficacy and safety of ambrisentan in a subgroup of connective tissue disease (CTD) patients with PAH.

**Methods:**

In this open-label, single-arm study, patients received ambrisentan 5 mg once daily for 12 weeks, followed by 12-week dose titration period (dose up to 10 mg). Efficacy endpoints included change from baseline in exercise capacity (measured by 6-min walk test [6MWT]), N-terminal pro B type natriuretic peptide (NT-proBNP) plasma levels, WHO Functional Class (FC) and Borg Dyspnoea Index (BDI) scores from baseline to weeks 12 and 24. Safety endpoints included time to clinical worsening and incidence of adverse events (AEs).

**Results:**

In total, 71 Chinese patients with CTD-PAH were included in this analysis. Ambrisentan treatment significantly improved exercise capacity (6MWT) from baseline (mean: 366.4 m) to week 12 (63.8 m, *p* < 0.001) and week 24 (73.2 m, *p <* 0.001). A significant reduction in NT-proBNP levels was observed from baseline (mean: 1837.5 ng/L) to week 12 (− 1156.8 ng/L, *p* < 0.001) and week 24 (− 1095.5 ng/L, *p <* 0.001). BDI scores decreased significantly at week 12 (− 0.6, *p <* 0.001) and week 24 (− 0.4, *p* = 0.002) from baseline (mean: 2.7). The WHO FC improved in 29 (40.8%) and 34 (47.9%) patients at weeks 12 and 24, respectively. Adverse events were reported in 52 (73.2%) patients. One patient (1.4%) experienced clinical worsening at week 24.

**Conclusion:**

Ambrisentan showed significant improvement in exercise capacity and no clinical worsening in the majority of Chinese patients with CTD-PAH in the 24-week treatment period. The AEs observed in the CTD-PAH subgroup were consistent with the known safety profile of ambrisentan in the overall Chinese PAH population.

**Trial registration:**

ClinicalTrial.gov Identifier, https://clinicaltrials.gov/, NCT01808313 Registration date (first time): February 28, 2013.

## Background

Pulmonary arterial hypertension (PAH) is a frequent and severe complication in patients with connective tissue diseases (CTD), a group of disorders which adversely affects the cardiac and the respiratory system. It is also known to have non-respiratory complications, such as renal disorders [1]. CTD is characterised by vascular injury, autoimmunity, tissue inflammation and organ dysfunction including systemic sclerosis (SSc), systemic lupus erythematosus (SLE) and mixed CTD [2]. PAH affects approximately 3 to 13% of CTD patients (CTD-PAH), and it is one of the leading causes of death [3–5]. A recent registry in China has reported that out of all patients diagnosed with PAH, 43% had CTD-PAH [6]. High doses of steroids and immune suppressants have demonstrated beneficial effect in the treatment of CTD-PAH [7]. The use of vasodilators has been recommended in WHO functional class II to class IV patients [8]. Since immunosuppressive therapy does not provide long-term benefits, it is recommended to use vasodilators in combination with immunosuppressants for the treatment of CTD-PAH [1, 9, 10].

Ambrisentan, a vasodilator, which is an endothelin-A (ET_A_) receptor-selective antagonist. It is approved for the treatment of patients with PAH (World Health Organisation [WHO] functional class [FC] II or III symptoms) at oral doses of 5 and 10 mg once daily. The efficacy of ambrisentan, both alone and in combination, has been evaluated in previous studies in patients with CTD-PAH and the results have demonstrated an improvement in exercise capacity [11, 12] and freedom from clinical worsening in 80% of the patients at 1 year [11]. These studies included higher proportion of Caucasians and patients of Asian ethnicity were very limited.

The available data suggest that patients with CTD-PAH may have an attenuated response to short-term treatment than other forms of PAH [13], though they have a similar response to long-term treatment [14]. This could be explained by the heterogeneity in the treatment response in patients with PAH and wide variety of significant comorbidities such as rheumatoid arthritis, dermatomyositis, polymyositis, interstitial lung disease, limited mobility and deconditioning commonly seen in patients with CTD-PAH [15, 16]. In addition to the observed heterogeneity in treatment response, there is insufficient data in the existing literature for CTD-PAH subgroup that necessitate the understanding of specific therapeutic needs in managing individuals with different ethnicities. In the recent study conducted in Chinese patients with PAH, it was shown that ambrisentan improved exercise capacity, measured by 6-min walk test (6MWT) in the 24-week treatment period [17]. This study included about 53% patients with CTD at baseline, thus providing an opportunity to evaluate whether ambrisentan is efficacious in patients with CTD-PAH to the same extent as in the overall PAH population. The present post-hoc subgroup analysis was performed to evaluate the efficacy of ambrisentan in improving exercise capacity in Chinese patients with CTD-PAH. Additionally, we provided data on the patients with idiopathic PAH/heritable PAH (IPAH/HPAH) to explore the difference in response to treatment based on underlying disease.

## Methods

### Study design

An open-label, phase IIIb, single-arm study was conducted in China in patients with PAH between 21 December 2012 and 15 August 2014 (NCT01808313). The detailed study design and results of the study have been reported elsewhere [17]. This post-hoc analysis was conducted using a subgroup of patients with CTD-PAH and IPAH/HPAH. Briefly, eligible patients received oral 5 mg ambrisentan once daily for 12 weeks in the primary evaluation period, followed by a 12-week dose adjustment period, during which the dose of ambrisentan was titrated to 10 mg depending on patients’ dose tolerance [17].

All patients provided written informed consent before any study-specific procedure was performed. The study was conducted in accordance with International Conference on Harmonisation guidelines for Good Clinical Practice and the ethical principles laid down in the Declaration of Helsinki (2008). The study protocol was approved by independent ethics committee or institutional review board at each centre. The affiliations of all the ethics committees (IECs) that approved the study are available in the additional file. Anonymised individual participant data and study documents can be requested for further research from www.clinicalstudydatarequest.com.

### Patients

Patients aged 18–75 years who had a diagnosis of symptomatic or severe PAH (WHO FC II or III), as defined in Group 1 of the current treatment guidelines [18], were enrolled in the study. Patients were included if they performed the 6MWT with a minimum distance of 150 m and a maximum distance of 450 m, had a right heart catheterisation performed within 6 months of screening with mean pulmonary artery pressure ≥ 25 mmHg; pulmonary vascular resistance ≥240 dyne.sec.cm^− 5^; and pulmonary capillary wedge pressure or left ventricular end diastolic pressure ≤ 15 mmHg. Patients with serum transaminase values > 2 times upper limit of normal (ULN), serum bilirubin > 1.5 times ULN, or haemoglobin concentration < 10 g/dL at baseline were excluded from the study. CTD patients were in an inactive state after the treatment of steroid with or without immunosuppresants. The detailed inclusion and exclusion criteria of patients have been described previously [17].

### Study assessments

This subgroup analysis assessed all primary and secondary endpoints from the primary study except for echocardiography assessments. The primary efficacy endpoint was change from baseline to week 12 in the exercise capacity, as measured by 6MWT. Other efficacy endpoints included change in 6MWT from baseline to week 24 and change in N-terminal pro B type natriuretic peptide (NT-proBNP) plasma levels, WHO FC and Borg Dyspnoea Index (BDI) scores from baseline to weeks 12 and 24. Changes in 6MWT, WHO FC and BDI scores were assessed at every visit, scheduled at 4-week intervals and changes in NT-pro BNP levels were assessed at weeks 12 and 24. Additionally, the effect of ambrisentan on heart rate recovery (defined as difference in heart rate at the end of 6MWT and at 1/2/3 min after completion of the 6MWT) at 1 min (HRR_1min_), 2 min (HRR_2min_) and 3 min (HRR_3min_) post-exercise was assessed.

Time to clinical worsening of PAH (defined as time from baseline to first occurrence of death, lung transplantation, hospitalisation for PAH treatment, atrial septostomy or discontinuation of ambrisentan due to change to other PAH treatment) was assessed.

Safety and tolerability assessments included monitoring and recording of adverse events (AEs) and serious AEs (SAEs), laboratory assessments, liver function tests, 12-lead electrocardiogram (ECG) and vital sign measurements.

### Statistical analysis

Efficacy analyses were performed on the intent-to-treat (ITT) population, which consisted of all patients with CTD-PAH and IPAH/HPAH who received at least one dose of ambrisentan and had baseline and at least one post-baseline efficacy assessment.

The change from baseline in 6MWT was assessed using a paired t-test. Changes in plasma NT-proBNP levels and BDI were assessed using a Wilcoxon Signed-Rank test. WHO FC outcomes were summarised descriptively in both CTD-PAH and IPAH/HPAH subgroups and the comparison between the CTD-PAH and IPAH/HPAH subgroups was performed using Chi-square test. The change in 6MWT was compared between CTD-PAH and IPAH/HPAH subgroups using a 2-group t-test. The last observation carried forward (LOCF) method was used to impute missing data for 6MWT, WHO FC and BDI assessments.

Time to clinical worsening was reported using Kaplan-Meier estimates along with corresponding 95% confidence intervals. Overall safety data were summarised descriptively. The safety population included all patients with CTD-PAH and IPAH/HPAH who received at least one dose of ambrisentan. Statistical analyses were conducted using SAS® Version 9.4.

## Results

### Patient disposition and baseline characteristics

Of the 134 patients with PAH enrolled in the primary study [17], all received at least one dose of ambrisentan and were included in the safety analyses. One subject did not have post-baseline assessments, and thus 133 patients were included in the ITT population. A total of 71 patients (53.4% of the overall ITT population) had CTD-PAH at baseline, data from whom were used in the current subgroup analysis. Sixty-seven (94.4%) patients completed the study and four patients discontinued the study — two patients discontinued due to a protocol deviation, one discontinued due to protocol-defined stopping criteria and one discontinued due to an adverse event. The demographics and baseline characteristics of the CTD-PAH and IPAH/HPAH subgroups are summarised in Table 1. Among patients with CTD-PAH, the mean age of patients was 39.0 years; 97% of the patients were women. The WHO FC at baseline was predominantly class II or III. For the CTD-PAH subgroup, the mean duration of exposure of ambrisentan was 164.6 days. The CTD medications at baseline primarily included, hydroxychloroquine, glucocorticoids and immunosuppressants. All patients enrolled were inactive CTD with or without CTD medications. Of the 71 patients with CTD-PAH, data is available for 64 patients (SLE-CTD-PAH = 41 patients and Non SLE-CTD-PAH = 23 patients) in the supplementary Fig. 1, Table 1 and Table 2.

**Table 1:** Patient demographics and baseline characteristics (ITT population): post-hoc analysis

	Ambrisentan	
	CTD-PAH (***N*** = 71)	IPAH/HPAH (***N*** = 50)
Age (years), median (IQR)	40 (19–59)	40.6 (30–47)
Women, n (%)	69 (97.2)	36 (72.0)
Men	2 (2.8)	14 (28.0)
CTD medications, n (%)		
Hydroxychloroquine	29 (40.8)	–
Glucocorticoids	51 (71.8)	–
Immunosuppressants	27 (38.0)	–
BMI, kg/m^2^	21.2 (3.2)	22.2 (3.2)
6MWT, m	366.4 (60.4)	397.3 (48.1)
BDI score, n (%)	2.7 (1.3)	2.3 (1.5)
Class II	32 (45.1)	31 (62.0)
Class III	39 (54.9)	19 (38.0)
NT-ProBNP, ng/L	1837.5 (2209.6)	1281.3 (1140.6)
History of cardiovascular risk factors, n (%)	3 (4.2)	0

*BDI* Borg Dyspnoea Index, *BMI* basal metabolic index, *CTD* connective tissue disease, *HPAH* Heritable PAH, *IPAH* Idiopathic PAH, *IQR* inter quartile range, *ITT* intent-to-treat, *NT-ProBNP* N-terminal pro hormone B-Type Natriuretic Peptide, *6MWT* six-minute walk test

**Table 2:** Mean change from baseline in 6MWT, BDI scores, and NT-proBNP levels after treatment with ambrisentan (ITT population): post-hoc analysis

Efficacy measure	Time point	CTD-PAH(***N*** = 71)	IPAH/HPAH (***N*** = 50)	Between-group difference^$^
**6MWT, m (LOCF)**	Baseline	366.4 (60.4)	397.3 (48.1)	
	Change from baseline (Week 12)	63.8 (57.4)	45.5 (69.7)	−18.39
	*p* value	< 0.001^*^	< 0.001^*^	0.115^¥^
	Change from baseline (Week 24)	73.2 (84.6)	62.2 (81.9)	−10.97
	*p* value	< 0.001^*^	< 0.001^*^	0.478^¥^
**BDI score (LOCF)**	Baseline	2.7 (1.4)	2.3 (1.5)	
	Change from baseline (Week 12)	−0.6 (1.1)	−0.2 (1.9)	0.35
	*p* value	< 0.001^§^	0.427^§^	0.203^¶^
	Change from baseline (Week 24)	−0.4 (1.70)	−0.2 (1.96)	0.19
	*p* value	0.002^§^	0.138^§^	0.377^¶^
**NT-proBNP, ng/L (Observed data)**	Baseline	1837.5 (2209.6)	1281.3 (1140.6)	
	Change from baseline (Week 12)	−1156.8 (1735.4)	−450.3 (699)	706.49
	*p* value	< 0.001^§^	< 0.001^§^	0.007^¶^
	Change from baseline (Week 24)	−1095.5 (1587.7)	− 396.6 (738.5)	698.89
	*p* value	< 0.001^§^	< 0.001^§^	0.011^¶^

*BDI* Borg Dyspnoea Index, *CTD* connective tissue disease, *HPAH* Heritable PAH, *IPAH* Idiopathic PAH, *ITT* intent-to-treat, *NT-ProBNP* N-terminal pro hormone B-Type Natriuretic Peptide, *6MWT* six-minute walk test

### Efficacy

**Fig. 1:**
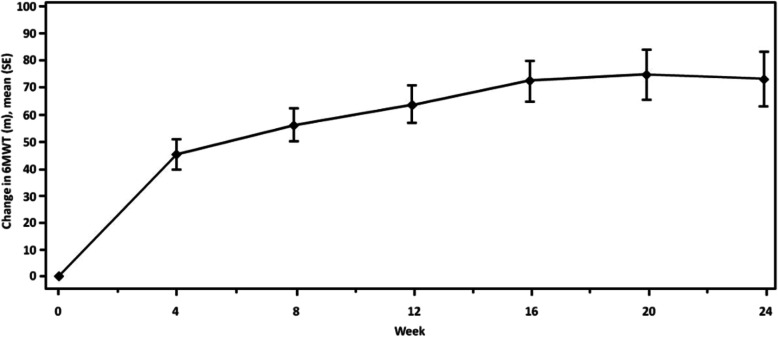
Improvement in 6MWT over 24 weeks following ambrisentan treatment (patients with connective tissue disease) (ITT population-LOCF): post-hoc analysis. 6MWT, 6-min walk test; ITT, intent-to-treat; LOCF, last observation carried forward

The time course of mean change from baseline in 6MWT in 24 weeks of treatment with ambrisentan is presented for patients with CTD-PAH in Fig. 1. In the CTD-PAH subgroup, a significant improvement of 63.8 m in 6MWT (baseline 366.4 m) was observed at 12 weeks and 73.2 m at 24 weeks of treatment with ambrisentan (*p* < 0.001 for both week 12 and week 24). The improvements in 6MWT were also significant for IPAH (*p* < 0.01). The improvement in 6MWT in the CTD-PAH subgroup was greater compared with the IPAH/HPAH subgroup resulting in a between-group difference of − 18.39 m at 12 weeks (*p* = 0.115) and − 10.97 m at 24 weeks (*p* = 0.478) (Table 2); however, the difference was not statistically significant.

In the CTD-PAH subgroup, an improvement of 0.6 in BDI score (baseline 2.7) was observed at 12 weeks (*p* < 0.001) and 0.4 at 24 weeks (*p* = 0.002) of treatment with ambrisentan. The improvement in BDI scores was greater in the CTD-PAH subgroup compared with the IPAH/HPAH subgroup with a between-group difference of 0.35 and 0.19 at weeks 12 and 24, respectively; however, the difference was not statistically significant (Table 2).

In the CTD-PAH subgroup, NT-proBNP levels decreased significantly with ambrisentan treatment at week 12 (− 1156.8 ng/L, *p <* 0.001) and week 24 (− 1095.5 ng/L, *p <* 0.001) compared to baseline (mean 1837.5 ng/L). The reductions in NT-proBNP at weeks 12 and 24 were significant in both CTD-PAH (*p <* 0.001) and IPAH/HPAH (*p <* 0.001) subgroups, but the reduction in NT-proBNP at weeks 12 and 24 was greater in the CTD-PAH subgroup compared with the IPAH/HPAH subgroup (Table 2).

**Fig. 2:**
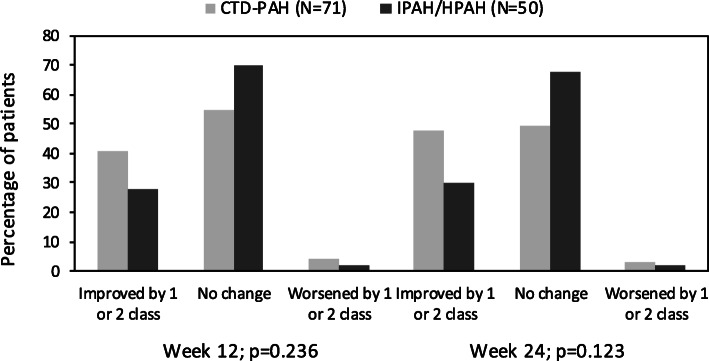
Patients categorized under WHO functional classification after treatment with ambrisentan (ITT population-LOCF): post-hoc analysis of CTD-PAH and IPAH/HPAH sub-groups. CTD, connective tissue disease; HPAH, Heritable PAH; IPAH, Idiopathic PAH; ITT, intent-to-treat; LOCF, last observation carried forward; PAH, pulmonary arterial hypertension; WHO, World Health Organisation

The WHO FC improved by one class in 29 (40.8%) patients at week 12 (improved from class III to II) and 34 (47.9%) patients at week 24 (class III to II, *n* = 31; class II to I, *n =* 3) in the CTD-PAH subgroup. Three patients at week 12 (worsened from class II to III) and two at week 24 (one patient worsened from class II to III and one patient worsened from class III to IV) showed deterioration in FC. A greater proportion of patients in CTD-PAH subgroup showed improvement in WHO FC at weeks 12 and 24 compared with IPAH/HPAH subgroup (Fig. 2).

In the CTD-PAH subgroup, a significant decrease in heart rate recovery was observed at 1 and 2 min post-exercise at 24 weeks of treatment with ambrisentan (change in heart rate recovery from baseline: − 3.0 and − 3.5 beats/min from baseline at 1 and 2 min post-exercise, *p* < 0.05).

### Safety

In the CTD-PAH subgroup, 52 (73.2%) patients experienced at least one AE (Table 3). One SAE (1.4%) was reported, which resulted in death. However, the death occurred due to cardiac failure and was not associated with ambrisentan treatment. The most common AE was flushing (*n* = 9 [12.7%]). Most of the AEs were mild (41%) or moderate (27%) in intensity. Six patients (8.5%) experienced SAEs that included bronchitis, lung infection, pneumonia, ascites, diarrhoea, cardiac failure and cystitis glandularis. None of these SAEs were considered to be related to ambrisentan treatment by the investigator. Elevation of AST and ALT was noted in 4.2 and 5.6% in the CTD-PAH and 11.8 and 9.8% in the IPAH/HPAH subgroups, respectively. Clinical worsening of PAH occurred in one (1.4%) patient, which resulted in death. Kaplan-Meier estimate of clinical worsening at week 24 was 98.5%. No significant changes were noted in the haematology parameters. No clinically relevant changes in vital signs, ECG and physical examination were observed.

**Table 3:** Summary of safety (safety population): post-hoc analysis

	Ambrisentan	
	CTD-PAH (***N*** = 71)	IPAH/HPAH (***N*** = 51)
Any AEs, n (%)	52 (73.2)	31 (60.8)
AEs related to study treatment	28 (39.4)	17 (33.3)
Discontinuation due to AE	1 (1.4)	2 (3.9)
Any serious AEs, n (%)	6 (8.5)	3 (5.9)
Deaths, n (%)	1 (1.4)	1 (2.0)
Most frequent (≥5% in either subgroup) AEs, n (%)		
Flushing	9 (12.7)	2 (3.9)
Oedema peripheral	7 (9.9)	8 (15.7)
Headache	6 (8.5)	0
Decreased appetite	5 (7.0)	1 (2.0)
Dizziness	5 (7.0)	1 (2.0)
Nausea	5 (7.0)	2 (3.9)
Alanine aminotransferase increased	4 (5.6)	5 (9.8)
Blood bilirubin increased	4 (5.6)	5 (9.8)
Blood glucose increased	2 (2.8)	3 (5.9)
Cough	4 (5.6)	2 (3.9)
Gamma glutamyltransferase increased	4 (5.6)	3 (5.9)
Aspartate aminotransferase increased	3 (4.2)	6 (11.8)

*AE* adverse event, *CTD* connective tissue disease, *HPAH* Heritable PAH, *IPAH* Idiopathic PAH, *PAH* pulmonary arterial hypertension

## Discussion

The results of this subgroup analysis showed that ambrisentan treatment significantly improved exercise capacity, as measured by the 6MWT, in Chinese patients with CTD-PAH as well as IPAH/HPAH. It was noteworthy that almost all the patients (97%) were women. Differential signalling, altered levels and altered metabolism of oestrogen have been reported to be the underlying factors responsible for the frequent occurrence of PAH in women than in men [19]. Almost all the patients in the CTD-PAH subgroup showed no clinical worsening up to 24 weeks of treatment with ambrisentan.

Following 12 weeks of treatment, the exercise capacity was significantly improved for patients in both CTD-PAH and IPAH/HPAH subgroups. The increase was noted further up to 24 weeks of treatment. The improvement seen in the CTD-PAH subgroup was more pronounced than that observed in the IPAH/HPAH subgroup; however, the between-group difference was not statistically significant. The improvement in exercise capacity in patients with CTD-PAH was also greater than that observed in the overall Chinese PAH population (53.6 m at week 12 and 64.4 m at week 24 from a baseline of 377.1 m) [17]. Additionally, the improvement in exercise capacity observed in this study in Chinese patients with CTD-PAH was substantially higher than that observed in a previous study in Caucasian patients with CTD-PAH [12, 20]. Although in this study, the magnitude of increase in 6MWT was greater in Chinese patients compared with Caucasians in other studies; this could be due to difference in disease characteristics of PAH associated with CTD between Chinese and Caucasian populations. The increased prevalence of SLE as underlying disease in Chinese patients compared with SSc in Caucasians may have resulted in better improvement in 6MWT in this subgroup analysis [12, 21].

The increased exercise capacity in 6MWT after treatment with ambrisentan was further supported by the significant improvement in BDI scores for patients with CTD-PAH but not for patients with IPAH/HPAH. In patients with CTD-PAH, the improvement seen in BDI scores was higher than the corresponding improvement seen in the overall population in the study (− 0.6 vs. -0.2 at week 12 and − 0.4 vs. -0.2 at week 24) indicating the pronounced effect of ambrisentan in CTD-PAH population [17]. These results are in agreement with previous studies, where improvement in BDI scores were observed at 12 and 24 weeks with both 5 mg and 10 mg doses [13, 22].

Clinical improvements were also observed in other efficacy parameters in this analysis. The NT-proBNP levels were significantly reduced in both CTD-PAH as well as IPAH/HPAH subgroups at 12 weeks of treatment, but the effect was more pronounced in the CTD-PAH subgroup. CTD-PAH and IPAH/HPAH subgroups did not show further improvements in plasma BNP levels, following 24 weeks of treatment. The decrease in NT-proBNP levels at 12 weeks of treatment with ambrisentan in patients with CTD-PAH was slightly higher to that observed in the overall PAH population in the study [17]. In contrast, there was 30 and 45% reduction in BNP levels at 5 mg and 10 mg doses of ambrisentan, respectively, in the ARIES-1 study [13].

Considering the progressive nature of disease, it is important to note the effect of ambrisentan on WHO FC. More than 40% of CTD-PAH patients and approximately 30% of IPAH/HPAH patients showed improvement in WHO FC in the present analysis. Among the overall PAH population in the study, 33.1 and 38.3% of the patients showed improvement in WHO FC at 12 and 24 weeks, respectively [17]. In the IPAH/HPAH subgroup, majority of the patients had not shown improvement in WHO FC, indicating less pronounced effect of ambrisentan in this subgroup. Previously, ambrisentan treatment has shown improvement in WHO FC in dose groups ranging from 2.5–10 mg in 12 weeks as well as 2 years [13, 22].

The heart rate recovery at 1 and 2 min post-exercise was faster following ambrisentan treatment at week 24. This finding was of significance as heart rate recovery post-exercise is known to be a strong predictor of clinical worsening and survival in patients with PAH [23, 24].

The majority of the AEs observed with ambrisentan were mild to moderate in intensity. The safety findings observed in the present analysis were similar to that in the earlier studies conducted in Western and overall Chinese PAH population [13, 17, 22, 24].

The results of the current subgroup analysis showed that the efficacy of ambrisentan is at least of a similar magnitude to, and possibly greater in, CTD/PAH than IPAH/HPAH. However, due to limited sample size of the subgroup and short-term treatment duration, definite conclusions cannot be derived. Nonetheless, results of the current analysis provide substantial evidence regarding the efficacy of ambrisentan in CTD-PAH population and warrant further research in this direction.

## Conclusions

Ambrisentan showed significant improvement in exercise capacity in Chinese patients with CTD-PAH. Most of the patients in the CTD-PAH subgroup showed either improvement in the WHO FC or no change; almost all patients in the CTD-PAH subgroup did not show any clinical worsening. The AEs observed in the CTD-PAH subgroup were consistent with the known safety profile of ambrisentan in the overall Chinese PAH population.

## Supplementary information

**Additional file 1: Supplementary Figure 1.** Change from baseline in WHO functional classification after treatment with ambrisentan (ITT population-LOCF): post-hoc analysis of SLE-CTD-PAH and Non SLE-CTD-PAH sub-groups. CTD, connective tissue disease; PAH, pulmonary arterial hypertension; SLE, systemic lupus erythematosus; WHO, World Health Organisation.

**Additional file 2: Supplementary Table 1.** Patient demographics and baseline characteristics (ITT population): post-hoc analysis.

**Additional file 3: Supplementary Table 2.** Mean change from baseline in 6MWT, BDI scores, and NT-proBNP levels after treatment with ambrisentan (ITT population): post-hoc analysis.

**Additional file 4.** Affiliations of all the ethics committees that approved the study.

## Data Availability

Anonymised individual participant data and study documents can be requested for further research from www.clinicalstudydatarequest.com.

## Abbreviations

ALT: Alanine aminotransfera
AST: Aspartate aminotransferase
AEs: Adverse events
BDI: Borg Dyspnoea index
CTD: Connective tissue disease
ECG: Electrocardiogram
ET_A_: Endothelin-a
FC: Functional class
HPAH: Heritable PAH
HRR: Heart rate recovery
IPAH: Idiopathic PAH
ITT: Intent-to-treat
LOCF: Last observation carried forward
NT-proBNP: N-terminal pro B type natriuretic peptide
PAH: Pulmonary arterial hypertension
SAEs: Serious AEs
SLE: Systemic lupus erythematosus
SSc: Systemic sclerosis
ULN: Upper limit of normal
WHO: World Health Organisation
6MWT: 6-min walk test
