# Supplementary material for: Efficacy and safety of ambrisentan in Chinese patients with connective tissue disease-pulmonary arterial hypertension: a post-hoc analysis
Source: BMC Cardiovasc Disord. 2020 Jul 17;20:339. doi: 10.1186/s12872-020-01591-1 (PMC7367256; doi:10.1186/s12872-020-01591-1)
Supplement: Supplementary file 1 — Additional file 1: Supplementary Figure 1. Change from baseline in WHO functional classification after treatment with ambrisentan (ITT population-LOCF): post-hoc analysis of SLE-CTD-PAH and Non SLE-CTD-PAH sub-groups. CTD, connective tissue disease; PAH, pulmonary arterial hypertension; SLE, systemic lupus erythematosus; WHO, World Health Organisation. [file 12872_2020_1591_MOESM1_ESM.docx]

**Supplementary Figure 1.** Change from baseline in WHO functional classification after treatment with ambrisentan (ITT population-LOCF): post-hoc analysis of SLE-CTD-PAH and Non SLE-CTD-PAH sub-groups

CTD, connective tissue disease; PAH, pulmonary arterial hypertension; SLE, systemic lupus erythematosus; WHO, World Health Organisation.
