# Supplementary material for: Efficacy and safety of ambrisentan in Chinese patients with connective tissue disease-pulmonary arterial hypertension: a post-hoc analysis
Source: BMC Cardiovasc Disord. 2020 Jul 17;20:339. doi: 10.1186/s12872-020-01591-1 (PMC7367256; doi:10.1186/s12872-020-01591-1)
Supplement: Supplementary file 2 — Additional file 2: Supplementary Table 1. Patient demographics and baseline characteristics (ITT population): post-hoc analysis. [file 12872_2020_1591_MOESM2_ESM.docx]

## Supplementary Table 1. Patient demographics and baseline characteristics (ITT population): post-hoc analysis

|  | Ambrisentan | |
| --- | --- | --- |
|  | SLE-CTD-PAH (n=41) | Non SLE-CTD-PAH (n=23) |
| Age (years), median (IQR) | 39.2 (29.4-44.2) | 42.4 (37.1-49.0) |
| Women | 40 | 22 |
| Men | 1 | 1 |
| BMI, kg/m^2^, mean (SD) | -21.29 (3.12) | -21.49 (3.45) |
| 6MWT, m, mean (SD) | 372.52 (59.7) | 370.89 (59.3) |
| BDI score, mean (SD) | -2.5 (1.0) | -2.2 (1.0) |
| WHO FC |  |  |
| Class II | 20 | 10 |
| Class III | 21 | 13 |
| NT-ProBNP, ng/L, mean (SD) | 1965.9 (2193.5) | 1250.0 (1940.4) |
| History of cardiovascular risk factors, n (%) | - | - |
| BDI, Borg dyspnoea index; BMI, basal metabolic index; CTD, connective tissue disease; ITT, intent-to-treat; NT-ProBNP, N-terminal pro hormone B-Type Natriuretic Peptide; PAH, pulmonary arterial hypertension; SLE, systemic lupus erythematosus; WHO FC, World Health Organization functional class; 6MWT, six-minute walk test | | |
