# Supplementary material for: Efficacy and safety of ambrisentan in Chinese patients with connective tissue disease-pulmonary arterial hypertension: a post-hoc analysis
Source: BMC Cardiovasc Disord. 2020 Jul 17;20:339. doi: 10.1186/s12872-020-01591-1 (PMC7367256; doi:10.1186/s12872-020-01591-1)
Supplement: Supplementary file 3 — Additional file 3: Supplementary Table 2. Mean change from baseline in 6MWT, BDI scores, and NT-proBNP levels after treatment with ambrisentan (ITT population): post-hoc analysis. [file 12872_2020_1591_MOESM3_ESM.docx]

**Supplementary Table 2. Mean change from baseline in 6MWT, BDI scores, and NT-proBNP levels after treatment with ambrisentan (ITT population): post-hoc analysis**

| **Efficacy measure** | **Time point** | **SLE-CTD-PAH (N=41)** | **Non SLE-CTD-PAH (N=23)** | **Between-group difference**^$^ |
| --- | --- | --- | --- | --- |
| **6MWT, m (LOCF)** | Baseline | 372.5 (59.7) | 370.9 (59.3) |  |
|  | Change from baseline (Week 12) | 69.3 (44.4) | 47.0 (67.7) | -22.4 |
|  | p value | <0.001^*^ | 0.003^*^ | 0.16^¥^ |
|  | Change from baseline (Week 24) | 78.3 (79.7) | 44.4 (96.4) | -33.9 |
|  | p value | <0.001^*^ | 0.038^*^ | 0.14^¥^ |
| **BDI score (LOCF)** | Baseline | -2.51 (1.03) | -2.20 (0.99) | - |
|  | Change from baseline (Week 12) | -0.426 (0.98) | -0.543 (0.99) | -0.12 |
|  | p value | 0.008 | 0.015 | 0.734 |
|  | Change from baseline (Week 24) | -0.585 (1.10) | -0.152 (1.36) | 0.43 |
|  | p value | 0.001 | 0.62 | 0.254 |
| **NT-proBNP, ng/L (Observed data)** | Baseline | 1965.9 (2193.5) | 1250.0  (1940.4) |  |
|  | Change from baseline (Week 12) | -1170.6 (1668.4) | -892.8 (1854.4) | 277.7 |
|  | p value | <0.001^§^ | 0.001 | 0.35^¶^ |
|  | Change from baseline (Week 24) | -1139.28 (1626.5) | -812.636 (1786.6) | 326.64 |
|  | p value | <0.001^§^ | 0.01^§^ | 0.213^¶^ |
| BDI, Borg dyspnoea index; CTD, connective tissue disease; ITT, intent-to-treat; NT-ProBNP, N-terminal pro hormone B-Type Natriuretic Peptide; PAH, pulmonary arterial hypertension; SLE, systemic lupus erythematosus; WHO FC, World Health Organization functional class; 6MWT, six-minute walk test | | | | |
